# Health burden and economic loss attributable to ambient PM_2.5_ in Iran based on the ground and satellite data

**DOI:** 10.1038/s41598-022-18613-x

**Published:** 2022-08-23

**Authors:** Sasan Faridi, Reza Bayat, Aaron J. Cohen, Ensieh Sharafkhani, Jeffrey R. Brook, Sadegh Niazi, Mansour Shamsipour, Heresh Amini, Kazem Naddafi, Mohammad Sadegh Hassanvand

**Affiliations:** 1 Center for Air Pollution Research (CAPR), Institute for Environmental Research (IER), Tehran University of Medical Sciences, 8th Floor, No. 1547, North Kargar Avenue, Tehran, Iran; 2 Department of Environmental Health Engineering, School of Public Health, Tehran University of Medical Sciences, Tehran, Iran; 3 Tehran Urban Planning and Research Center, Tehran Municipality, Tehran, Iran; 4 Institute for Health Metrics and Evaluation, University of Washington, Seattle, WA USA; 5 School of Public Health, Boston University, Boston, MA USA; 6 Health Effects Institute, Boston, MA USA; 7 School of Environment, College of Engineering, University of Tehran, Tehran, Iran; 8 Dalla Lana School of Public Health, University of Toronto, Toronto, ON M5T 1P8 Canada; 9 International Laboratory for Air Quality and Health (ILAQH), School of Earth and Atmospheric Sciences, Faculty of Science, Queensland University of Technology (QUT), Brisbane, 4001 Australia; 10 Department of Research Methodology and Data Analysis, Institute for Environmental Research (IER), Tehran University of Medical Sciences, Tehran, Iran; 11 Department of Public Health, University of Copenhagen, Copenhagen, Denmark; 12 Department of Environmental Health, Harvard T.H. Chan School of Public Health, Boston, MA USA

**Keywords:** Environmental impact, Environmental sciences

## Abstract

We estimated mortality and economic loss attributable to PM_2·5_ air pollution exposure in 429 counties of Iran in 2018. Ambient PM_2.5_-related deaths were estimated using the Global Exposure Mortality Model (GEMM). According to the ground-monitored and satellite-based PM_2.5_ data, the annual mean population-weighted PM_2·5_ concentrations for Iran were 30.1 and 38.6 μg m^−3^, respectively. We estimated that long-term exposure to ambient PM_2.5_ contributed to 49,303 (95% confidence interval (CI) 40,914–57,379) deaths in adults ≥ 25 yr. from all-natural causes based on ground monitored data and 58,873 (95% CI 49,024–68,287) deaths using satellite-based models for PM_2.5_. The crude death rate and the age-standardized death rate per 100,000 population for age group ≥ 25 year due to ground-monitored PM_2.5_ data versus satellite-based exposure estimates was 97 (95% CI 81–113) versus 116 (95% CI 97–135) and 125 (95% CI 104–145) versus 149 (95% CI 124–173), respectively. For ground-monitored and satellite-based PM_2.5_ data, the economic loss attributable to ambient PM_2.5_-total mortality was approximately 10,713 (95% CI 8890–12,467) and 12,792.1 (95% CI 10,652.0–14,837.6) million USD, equivalent to nearly 3.7% (95% CI 3.06–4.29) and 4.3% (95% CI 3.6–4.5.0) of the total gross domestic product in Iran in 2018.

## Introduction

PM_2.5_ has been recognized as the leading environmental cause of respiratory-, cardiovascular, and cancer-related deaths worldwide^1–4^. Based on the Global Burden of Disease (GBD) study, the number of deaths and crude death rate per 100,000 population attributable to ambient PM_2.5_ exposures in Iran increased from 36,379 (95% CI 32,140–40,502) and 47 (95% CI 42–53) in 2010 to 41,742 (95% CI 36,156–47,037) and 49 (95% CI 43–56) in 2019, whereas the figure for age-standardized death rate per 100,000 population declined from 74 (95% CI 65–83) to 63 (95% CI 55–71) (https://vizhub.healthdata.org/gbd-compare/). Also, exposure to ambient PM_2.5_ reduced the average of life expectancy at birth for Iran in the range of 0.76–1.02 years in 2016^5^. Recent estimates predict that global PM_2.5_-related deaths may double by 2050^6^. Current estimates also suggest that global economic loss attributable to health effects of ambient PM_2.5_ exposure is substantial and may increase until 2030^7–9^. It has been reported that 99% of the global population remain exposed to annual PM_2.5_ levels above the updated World Health Organization Air Quality Guidelines (WHO AQGs: 5 μg m^−3^)^10^. Though ambient PM_2.5_ concentrations have declined in high-income countries, levels in many low- and middle-income countries (LMICs) remain unchanged or have increased due to largely the lack of strong air quality management policies^11–13^.

Ambient air pollution in Iran as a LMIC has been considered as one of the most important public health risk factor by the government, policy-makers, health authorities, national and even international public health bodies as well as environmental health researchers^14–17^. National investigations have been conducted to estimate the health effects of ambient PM_2.5_ air pollution in Iran^16,17^. Little, however, is known about both health effect and economic loss attributable to ambient PM_2.5_ in all Iranian counties. In this context, we designed this study to estimate PM_2.5_-related health, namely all-cause and cause-specific mortality, such as ischemic heart disease (IHD), cerebrovascular disease (stroke), chronic obstructive pulmonary disease (COPD), lung cancer (LC) and lower respiratory infection (LRI), and YLL and economic loss for 429 counties and 31 provinces of Iran in 2018.

## Methods

We used two different but complementary approaches to estimate long-term exposure to ambient PM_2.5_ and its impact on mortality and economic loss. We initially obtained ambient PM_2.5_ data from all the available ground-based ambient air quality monitoring stations (AQMSs) across Iran, processed and validated the hourly PM_2.5_ concentrations and then estimated human exposure. We also used the high-resolution satellite-derived PM_2.5_ data from the GBD study to estimate human exposure. We then obtained the population data and the national baselines of all-cause mortality and cause specific deaths to estimate the health burden attributable to ambient PM_2.5_ exposures by using the GEMM function. After that, we estimated the crude death rate and age-standardized death rate per 100,000 population associated with PM_2.5_ air pollution exposure in the Iranian provinces and counties on the basis of the WHO standard population (https://www.who.int/healthinfo/paper31.pdf). We estimated the economic loss due to ambient PM_2.5_-related health effects by using two well-documented methods including the value of statistical life (VSL) and the value of life year (VOLY). Finally, we compared our findings that were estimated based on the ground-monitored and satellite-based PM_2.5_ data.

### Study domain

Iran is a country located in the Eastern Mediterranean region and is characterized by diverse geo-climatic areas, ranging from arid and semi-arid to subtropical along the Caspian coast and the northern forests. With nearly 81 million inhabitants in 31 provinces and 429 counties, Iran is the world's 17th most populous country in 2018. Spanning 1,648,195 km^2^, it is the second largest country in the Middle East and the 17th largest in the world^15^ (details in Supplementary Text 1).

### Estimation of human exposures based on the ground-monitored and satellite-based PM2.5 data

**Figure 1:**
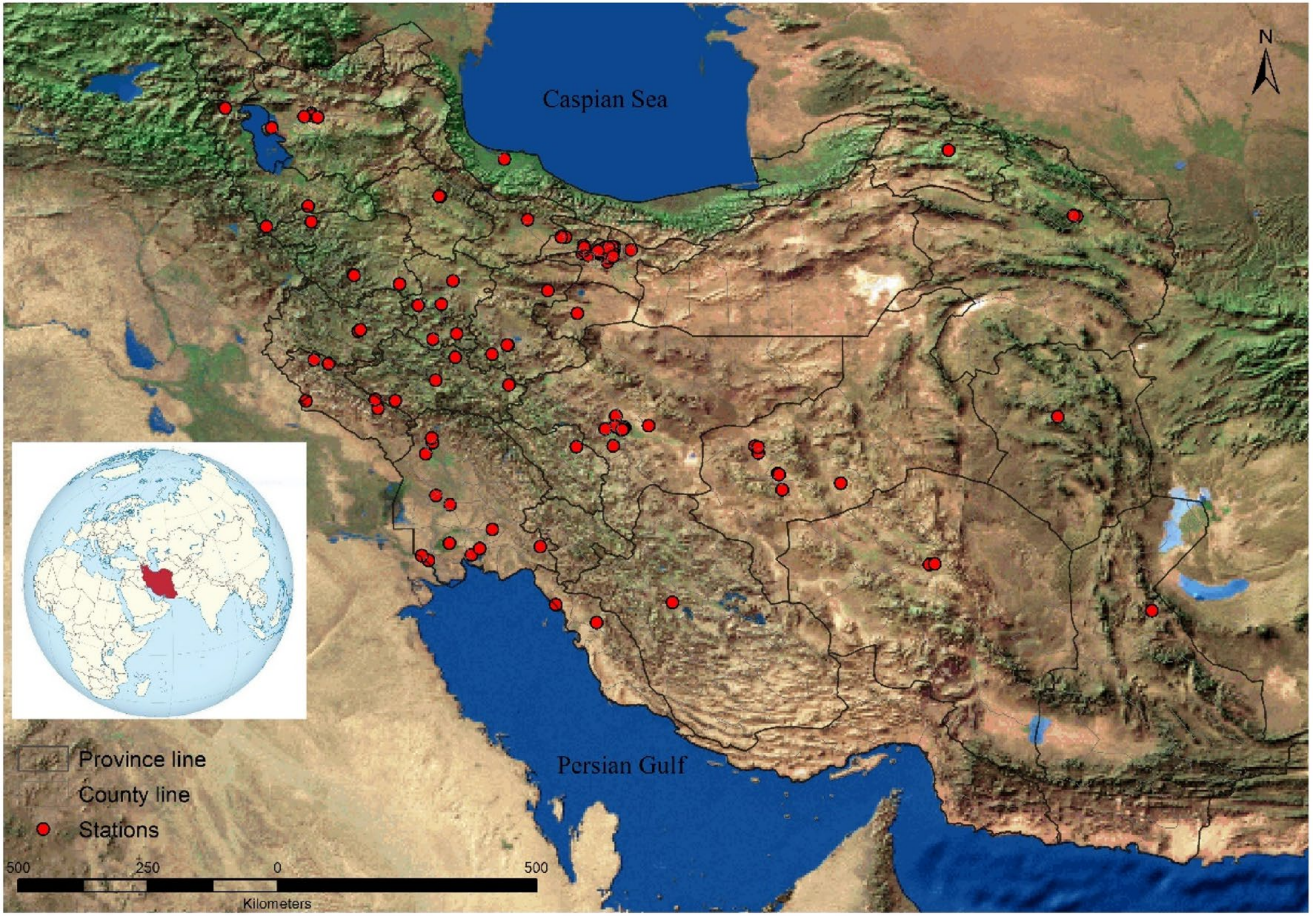
Spatial distribution of AQMSs across Iranian provinces in 2018 (this figure was generated by ArcGIS v10.7.0.10450).

We obtained the real-time hourly data on ambient PM_2.5_ concentrations from January 1, 2018, to December 31, 2018, from all the available AQMSs provided by the Iranian Department of Environment (DoE) and Tehran Air Quality Control Company (TAQCC). There were 229 AQMSs throughout 70 Iranian counties located in 25 provinces (out of 31) of Iran (Fig. 1). Of the 229 AQMSs, 147 monitored the ambient PM_2.5_ levels of Iranian counties for the year 2018 (Supplementary Text 2). To remove any inconsistency which might arise owing to operation disruptions at the AQMSs (e.g. routine maintenance activities, communication failures and power outages), hourly data were pre-processed and validated by using Z scores approach (Supplementary Text 3)^18–20^. Prior to the estimation of the health effects and economic loss of PM_2.5_ for each of 429 counties, the missing hourly PM_2.5_ data for the included AQMSs were estimated by using the hourly ratio of PM_2.5_/PM_10_ according to the available hourly data during the year of study, 2018 (Supplementary Text 4). As mentioned above, we also used the high-resolution satellite-derived PM_2.5_ data from the GBD study 2017 and 2019 (the average of both years was used for 2018)^3,21^. Though full details reported elsewhere, note that he GBD study provides datasets for ambient PM_2·5_ concentrations at a 0·1° × 0·1° resolution (~ 11 × 11 km) that these datasets include approximately 15,800 grids for Iran. To estimate human exposures to PM_2.5_ for each of 429 Iranian counties, we averaged the ambient PM_2·5_ concentrations of grids that were within the border of each of counties. Then, we estimated the health effects and economic loss of exposure to ambient PM_2·5_ for each of counties using the figures averaged. We calculated the population-weighted mean (PWM) PM_2·5_ concentrations at national level based on both ground-monitored and satellite-based ambient PM_2·5_ data using the following equation^20,22^.

$${\mathrm{PWM}} \, {\mathrm{at}} \, {\mathrm{national}}-\mathrm{level}= \frac{{\sum }_{\mathrm{i}=1}^{\mathrm{n}}\left({\mathrm{PM}}_{2.5\mathrm{i}}\times {\mathrm{Pop}}_{\mathrm{i}}\right)}{\sum_{\mathrm{i}=1}^{\mathrm{n}}{\mathrm{Pop}}_{\mathrm{i}}}$$

where PM_2.5_ is the mass concentration of ambient PM_2.5_. Pop is the population number. The suffixes i show each of investigated counties and n is the number of all counties within each province and country.

### Mortality baseline and population data

We obtained the population data within each population group for Iranian counties from the Statistical Centre of Iran (https://www.amar.org.ir/english/Population-and-Housing-Censuses). Moreover, national baselines of all-cause mortality and cause specific mortalities (stroke, IHD, COPD, LC and LRI) for each age group were obtained from the Institute for Health Metrics and Evaluation (IHME), GBD web-system (http://ghdx.healthdata.org/gbd-results-tool).

### Assessing PM_2.5_-related burden of disease

We estimated the ambient PM_2.5_-related health effects (e.g., all-cause mortality and five specific causes of death including IHD, stroke, COPD, LC and LRI) using the BenMAP-CE v1.5 software based on the concentration–response function developed by Burnet et al.^23,24^ as follow:

- Global Exposure Mortality Model (GEMM):
  $$\mathrm{GEMM }\left(\mathrm{z}\right)=\mathrm{exp}\left\{\frac{\mathrm{\theta ln}(\frac{\mathrm{z}}{\mathrm{\alpha }}+1)}{1+\mathrm{exp}(-\frac{\mathrm{z}-\upmu }{\upnu })}\right\}$$

θ, α, μ, and ν present the shape of response function estimating all-cause mortality and cause specific deaths. Where, Z presents the annual average concentration of ambient PM_2.5_ for each city; Z_cf_ known counterfactual concentration is the lowest observed concentration in any of the 41 cohorts (2.4 µg m^−3^) from Canada, United States, Europe and Asia below which no change in the hazard ratio is assumed. The following equation is used to estimate the health burden:

$$\mathrm{NPM}=\mathrm{BI }\times \mathrm{PAF}$$

where NPM denotes the number of mortalities in age group caused by ambient PM_2.5_ for all-cause mortality and cause specific mortalities; BI (baseline incidence) is a certain BI for each of health endpoints within each of population groups and PAF is the population attributable fraction computed by multiplying the population (P) and the relative risks (RRs, based on the GEMM function), as shown in following equation:

$$\mathrm{PAF}= \frac{\mathrm{RR}-1}{\mathrm{RR}}$$

### YLL

In the present study, the number of YLL was limited to all-cause mortality from long-term exposure to ambient PM_2.5_ for adults aged ≥ 25 years. We calculated YLL for Iran in 2018 using the following equation^17,25^:

$${\mathrm{YLL}}_{\mathrm{i}}={\mathrm{LE}}_{\mathrm{i}}\times {\mathrm{NPM}}_{\mathrm{i}}$$

where LE_i_ and NPM_i_ are the remaining life expectancy and the number of deaths in age group i, respectively. The YLL was the sum of YLL for all age groups. In this analysis, we used life expectancy tables for Iran from the IHME, GBD study (https://vizhub.healthdata.org/gbd-compare/arrow).

### Economic valuation estimates

Two approaches (including VSL and VOLY) were used to estimate the economic loss due to ambient air pollution-related health effects^8,16,25–27^. We used the VSL information reported for Co-operation and Development (OECD) countries (the mean VSL for OECD countries equal to 3.833 million USD) because of the lack of national studies on estimating VSL in Iran^16,25^. We, therefore, adjusted/calculated VSL for our study as follow.

$${\mathrm{VSL}}_{\mathrm{Iran}}={\mathrm{VSL}}_{\mathrm{OECD}}\times {\left(\frac{{\mathrm{GDP}}_{\mathrm{Iran}}}{{\mathrm{GDP}}_{\mathrm{OECD}}}\right)}^{\mathrm{b}}$$

Here, GDP_Iran_ and GDP_OECD_ represent the GDP per capita for Iran and OECD countries, respectively. Also, b represents the income elasticity of the VSL for different countries, ranging from 1.0 to 1.4 for low-income and middle-income countries^16,25^. We used 1.2 (as average of 1.0–1.4) for b in the present study^16,25^ because the average transfer error is smallest when the central elasticity value of 1.2 is considered^28^. As reported previously^19^, GDP for Iran and OECD countries were 3598 and 39,339 USD in 2018, respectively. As a result, based on the aforementioned assumptions, the VSL_Iran_ was equal to 217,282 USD for the year 2018. Lastly, the economic costs of health burden was evaluated by multiplying the number of all cause and cause specific mortalities attributable to ambient PM_2.5_ and adjusted VSL for Iran, as shown in the following equation:

$${\mathrm{Economic}} \, {\mathrm{burden} } = {\mathrm{VSL}}_{\mathrm{Iran}}\times {\mathrm{Mortality}}_{\mathrm{county}}$$

where Mortality_county_ is the number of all-cause and cause-specific deaths attributable to long-term exposure to ambient PM_2.5_ for each of 429 Iranian counties. Our second approach to valuing premature deaths was to calculate the economic value of YLL due to ambient PM_2.5_ exposures. Empirical values of VOLY have been either determined using WTP studies or approximated as a multiple of GDP per capita^25^. For our analysis, we used a VOLY equal to one GDP per capita as follow^30^:

$${\mathrm{VOLY}}_{\mathrm{Iran}}={\mathrm{YLL}}_{\mathrm{i}} \times {\mathrm{GDP}}_{\mathrm{Iran}}$$

## Results

### Overview of ambient PM_2.5_ concentration and its health and economic impacts at national level in Iran

**Figure 2:**
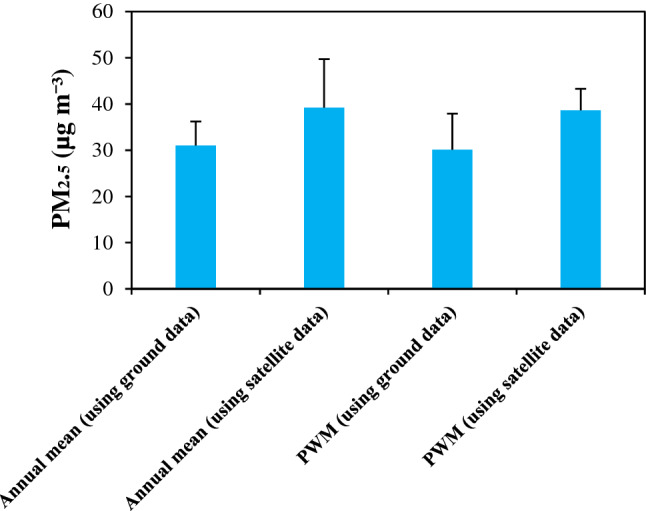
Annual mean and population-weighted mean (PWM) concentrations for ambient PM_2.5_ in Iran, 2018, based on ground and satellite data.

Figure 2 shows the annual mean PM_2.5_ concentrations for Iran in 2018. The average (± standard deviation) of PM_2.5_ concentrations based on the AQMSs and satellite measurements were 31.0 (± 5.2) and 39.2 (± 10.5) μg m^−3^, respectively, while the PWM PM_2·5_ concentrations for both approaches were 30.1 (± 7.8) and 38.6 (± 4.7) μg m^−3^ in 2018. The average difference between both datasets was nearly 8.0 μg m^−3^. Table 1 provides the results on all cause and cause specific mortalities due to ambient PM_2.5_ exposures, estimating based on ground and satellite-based datasets. The number of all-cause mortality attributable to long-term exposure to ambient PM_2.5_ using ground-monitored data was 49,303 (95% CI 40,914–57,379), whereas this figure stood at 58,873 (95% CI 49,024–68,287) using satellite-based data. As shown in Table 1, the highest and lowest number of cause specific deaths due to ambient PM_2.5_ in Iran was found for IHD [24003 (95% CI 22,051–25,887)] and LC [1884 (95% CI 1180–2502)], respectively when applying ground-monitored data. Similar trend can be reported while using satellite data. PM_2.5_-related all-cause, IHD, stroke, LRI, COPD and LC deaths were responsible for about 15%, 26%, 17%, 49%, 23% and 26% of those deaths in Iran when applying ground-monitored data. Among five specific causes of death, PM_2.5_-related IHD and stroke caused about 61% (49% for IHD and 12% for stroke) of total PM_2.5_-related deaths in Iran. Considering both approaches, the number of all cause and cause specific deaths attributable to ambient PM_2.5_ exposures has increased with age groups (Figure S2). More than 68% of the total deaths attributable to PM_2.5_ were from the elderly group (aged 65 or more); nearly 31% in the age group 65–79 years and 37–38% in the age group of 80 and up. Crude death and age-standardized death rates per 100 000 population attributable to ambient PM_2.5_ exposures in Iran were 97 (95% CI 81–113) and 125 (95% CI 104–145), respectively using ground-monitored approach, while these figures for satellite-based PM_2.5_ data arose to 116 (95% CI 97–135) and 149 (95% CI 124–173). The economic loss due to the total mortality attributable to ambient PM_2.5_ air pollution exposures for ground and satellite-based approaches were 10,712.6 (95% CI 8889.9–12,467.4) and 12,792.1 (95% CI 10,652.0–14,837.6) million USD, respectively in Iran (Table 2), equivalent to nearly 3.7% (95% CI 3.06–4.29) and 4.3% (95% CI 3.6–4.5.0) of the total GDP in the country (https://countryeconomy.com/gdp/iran) in 2018. In addition, the economic loss in Iran due to the total mortality attributable to ambient PM_2.5_ air pollution exposures was nearly 133 and 160 USD per capita per year in 2018 when applying ground and satellite-based approaches.

**Table 1:** Number of all cause and cause specific mortalities due to ambient PM_2.5_ exposure in Iran (2018).

Health outcomes (Mortality)	Attributable deaths (95% CI)	PAF (95% CI)	Crude death rate (95% CI)	Age-standardized death rate (95% CI)
**For ground-based data**				
All-cause	49,303 (40,914–57,379)	14.9 (12.3–17.3)	97 (81–113)	125 (104–145)
IHD	24,003 (22,051–25,887)	25.9 (23.8–28.0)	47 (43–51)	60 (55–65)
Stroke	6022 (3062–8657)	17.4 (8.8–25.0)	12 (6–17)	15 (8–22)
LRI	3685 (2089–4815)	49.2 (27.9–64.3)	7 (4–9)	9 (5–12)
COPD	2479 (1250–3543)	23.3 (11.8–33.3)	5 (2–7)	6 (3–9)
LC	1884 (1180–2502)	25.9 (16.2–34.4)	4 (2–5)	5 (3–6)
**For satellite-based data**				
All-cause	58,873 (49,024–68,287)	17.7 (14.8–20.6)	116 (97–135)	149 (124–173)
IHD	28,074 (25,864–30,193)	30.3 (27.9–32.6)	55 (51–60)	71 (65–76)
Stroke	7456 (3845–10,581)	21.5 (11.1–30.6)	15 (8–21)	19 (10–27)
LRI	4121 (2392–5276)	55.0 (31.9–70.4)	8 (5–10)	10 (6–13)
COPD	2957 (1513–4170)	27.8 (14.2–39.2)	6 (3–8)	8 (4–11)
LC	2259 (1434–2963)	31.1 (19.7–40.7)	4 (3–6)	6 (4–8)

**Table 2:** Number of all-cause mortality, crude and age-standardized death rate (for age-group ≥ 25 years old) and economic loss (× 10^6^ USD) in the 31 provinces of Iran due to PM_**2·5**_ in 2018 based on both ground-monitored and satellite-based PM_2.5_ data.

Provinces	Population aged ≥ 25 year (ratio to all population)	Mean concentration (min–max), µg m^−3^	Province-level mean concentrations compared with WHO AQG (ratio)	Attributable deaths (95% CI)	Crude death rate (95% CI)	Age-standardized death rate (95% CI)	Economic loss (95% CI)
**Based on the ground-monitored PM**_**2.5**_** data**							
Tehran	9,131,772 (67.0)	31.2 (24.6–39.1)	6.2	9899 (8224–11,508)	108 (90–126)	136 (113–158)	2150.9 (1787.0–2500.5)
Razavi Khorasan	3,908,350 (58.3)	29.0 (26.2–31.6)	5.8	3795 (3149–4416)	97 (81–113)	128 (106–149)	824.5 (684.2–959.6)
Isfahan	3,427,998 (65.4)	28.5 (23.8–35.1)	5.7	3509 (2909–4088)	102 (85–119)	122 (101–142)	762.5 (632.1–888.2)
Khuzestan	2,772,061 (57.3)	52.6 (43.2–66.1)	10.5	3375 (2826–3893)	122 (102–140)	189 (158–218)	733.3 (614.0–846.0)
Fars	3,144,791 (63.4)	31.5 (27.3–38.6)	6.3	3294 (2736–3830)	105 (87–122)	135 (112–157)	715.7 (594.5–832.3)
East Azerbaijan	2,534,087 (63.6)	23.7 (19.8–25.8)	4.7	2264 (1872–2645)	89 (74–104)	101 (83–118)	492.0 (406.7–574.7)
Mazandaran	2,265,027 (67.8)	24.4 (23.3–25.3)	4.9	2133 (1765–2489)	94 (78–110)	109 (90–127)	463.4 (383.5–540.8)
Gilan	1,773,027 (69.5)	23.4 (20.6–27.1)	4.7	1772 (1465–2070)	100 (83–117)	102 (85–120)	385.1 (318.4–449.8)
West Azerbaijan	1,982,994 (59.1)	24.7 (20.9–32.7)	4.9	1619 (1340–1889)	82 (68–95)	108 (89–126)	351.7 (291.1–410.4)
Kerman	1,841,477 (57.3)	26.3 (23.5–27.9)	5.3	1521 (1260–1774)	83 (68–96)	116 (96–135)	330.6 (273.8–385.4)
Alborz	1,852,037 (65.8)	25.8 (24.1–28.2)	5.2	1439 (1192–1678)	78 (64–91)	112 (93–131)	312.6 (258.9–364.6)
Kermanshah	1,267,973 (64.1)	33.2 (26.1–41.3)	6.6	1383 (1149–1607)	109 (91–127)	137 (114–159)	300.4 (249.6–349.2)
Hamadan	1,120,139 (63.6)	27.0 (19.4–33.8)	5.4	1143 (947–1333)	102 (84–119)	114 (94–133)	248.3 (205.7–289.6)
Lorestan	1,083,096 (60.7)	32.2 (19.6–44.5)	6.4	1056 (876–1229)	98 (81–114)	123 (102–143)	229.5 (190.3–267.1)
Markazi	940,999 (64.6)	24.5 (22.7–27.5)	4.9	927 (767–1082)	98 (81–115)	107 (89–125)	201.3 (166.6–235.1)
Golestan	1,129,935 (58.7)	26.4 (24.1–28.2)	5.3	905 (749–1054)	80 (66–93)	119 (99–139)	196.5 (162.8–229.1)
Sistan and Baluchestan	1,258,310 (43.2)	28.9 (28.3–30.5)	5.8	899 (746–1047)	71 (59–83)	124 (103–144)	195.3 (162.0–227.4)
Kurdistan	1,012,987 (61.7)	25.8 (21.0–30.4)	5.2	879 (728–1025)	87 (72–101)	113 (94–132)	191.0 (158.2–222.8)
Hormozgan	1,004,750 (53.9)	28.6 (26.6–31.2)	5.7	735 (609–856)	73 (61–85)	121 (101–141)	159.6 (132.4–185.9)
Qazvin	826,000 (63.2)	28.1 (25.2–30.2)	5.6	720 (598–839)	87 (72–102)	118 (98–137)	156.5 (129.8–182.3)
Qom	796,006 (59.2)	28.1 (25.0–30.0)	5.6	657 (544–765)	82 (68–96)	121 (101–141)	142.7 (118.3–166.2)
Ardabil	797,993 (61.9)	22.7 (21.9–23.4)	4.5	654 (541–764)	82 (68–96)	103 (85–120)	142.1 (117.5–165.9)
Yazd	696,041 (58.5)	26.6 (23.3–28.4)	5.3	649 (538–757)	93 (77–109)	119 (99–139)	141.1 (116.9–164.4)
Zanjan	1,852,037 (65.8)	25.7 (23.8–27.8)	5.1	625 (518–730)	93 (77–109)	112 (93–131)	135.9 (112.5–158.5)
Bushehr	722,304 (59.7)	36.3 (29.1–47.0)	7.3	614 (511–713)	85 (71–99)	140 (117–163)	133.4 (110.9–155.0)
Chaharmahal and Bakhtiari	573,613 (59.2)	33.5 (25.9–40.1)	6.7	593 (492–689)	103 (86–120)	133 (111–155)	128.8 (107.0–149.8)
North Khorasan	499,941 (56.4)	30.0 (28.6–31.6)	6.0	489 (406–569)	98 (81–114)	129 (107–150)	106.3 (88.2–123.7)
Kohgiluyeh and Boyer-Ahmad	418,919 (57.2)	42.3 (35.7–50.1)	8.5	459 (383–532)	110 (91–127)	159 (133–185)	99.7 (83.1–115.6)
South Khorasan	437,111 (54.9)	25.4 (23.3–27.0)	5.1	447 (370–522)	102 (85–119)	111 (92–130)	97.1 (80.4–113.3)
Ilam	369,998 (62.5)	42.7 (38.5–46.8)	8.5	430 (359–498)	116 (97–135)	168 (140–194)	93.5 (78.0–108.2)
Semnan	461,984 (63.1)	25.9 (23.5–27.6)	5.2	419 (347–489)	91 (75–106)	112 (93–131)	91.1 (75.4–106.3)
All provinces	50,721,726 (62.8)	30.0 (19.4–66.1)	6.0	49,303 (40,914–57,379)	97 (81–113)	125 (104–145)	10,712.6 (8889.9–12,467.4)
**Based on the satellite-based PM**_**2.5**_** data derived from the GBD study**							
Tehran	Note that the population data reported by Iranian governmental organizations was used to estimate health burden and economic losses in the provinces and counties of Iran	36.7 (32.1–40.7)	7.3	10,515 (8746–12,210)	115 (96–134)	145 (120–168)	2284.8 (1900.4–2653.1)
Isfahan		42.4 (33.1–51.3)	8.5	5015 (4189–5800)	146 (122–169)	174 (145–201)	1089.8 (910.3–1260.2)
Razavi Khorasan		39.2 (30.7–49.7)	7.8	4567 (3803–5296)	117 (97–136)	154 (128–179)	992.2 (826.4–1150.7)
Fars		39.9 (33.1–50.7)	8.0	3810 (3174–4417)	121 (101–140)	156 (130–181)	827.9 (689.7–959.8)
Khuzestan		58.4 (42.5–68.4)	11.7	3632 (3048–4180)	131 (110–151)	203 (170–234)	789.1 (662.3–908.2)
East Azerbaijan		28.3 (26.2–30.5)	5.7	2771 (2297–3228)	109 (91–127)	123 (102–143)	602.1 (499.2–701.4)
Mazandaran		30.6 (28.2–33.8)	6.1	2557 (2122–2975)	113 (94–131)	130 (108–152)	555.6 (461.1–646.4)
Gilan		28.7 (27.5–30.1)	5.7	2154 (1786–2509)	122 (101–142)	124 (103–145)	468.1 (388.1–545.1)
Kerman		37.8 (30.7–47.1)	7.6	2009 (1673–2330)	109 (91–127)	153 (127–177)	436.5 (363.5–506.2)
West Azerbaijan		29.5 (27.3–32.2)	5.9	1896 (1573–2207)	96 (79–111)	126 (105–147)	411.9 (341.7–479.5)
Alborz		33.5 (30.8–35.6)	6.7	1777 (1477–2064)	96 (80–111)	139 (115–161)	386.1 (320.9–448.6)
Kermanshah		39.4 (36.6–42.5)	7.9	1606 (1338–1861)	127 (106–147)	159 (132–184)	348.9 (290.8–404.4)
Sistan and Baluchestan		56.7 (40.6–87.6)	11.3	1407 (1180–1621)	112 (94–129)	194 (163–224)	305.7 (256.3–352.2)
Hamadan		33.4 (31.1–35.4)	6.7	1381 (1147–1605)	123 (102–143)	138 (114–160)	300.0 (249.2–348.7)
Lorestan		36.8 (31.5–44.9)	7.4	1235 (1027–1434)	114 (95–132)	144 (120–167)	268.3 (223.1–311.6)
Kurdistan		33.9 (31.1–37.5)	6.8	1087 (903–1263)	107 (89–125)	140 (116–163)	236.2 (196.3–274.4)
Markazi		30.0 (27.9–32.8)	6.0	1084 (899–1263)	115 (96–134)	126 (104–146)	235.6 (195.3–274.3)
Golestan		32.2 (30.4–34.1)	6.4	1066 (885–1239)	94 (78–110)	141 (117–164)	231.5 (192.4–269.2)
Yazd		45.9 (38.0–50.3)	9.2	969 (810–1120)	139 (116–161)	178 (149–206)	210.6 (176.0–243.4)
Bushehr		56.6 (47.0–67.6)	11.3	897 (753–1032)	124 (104–143)	205 (172–236)	194.8 (163.5–224.2)
Hormozgan		35.6 (31.0–44.6)	7.1	853 (709–991)	85 (71–99)	141 (117–164)	185.4 (154.1–215.3)
Ardabil		28.9 (27.8–31.0)	5.8	787 (653–917)	99 (82–115)	124 (103–144)	171.0 (141.8–199.2)
Qom		34.7 (31.4–36.9)	6.9	768 (639–892)	96 (80–112)	142 (118–165)	166.9 (138.7–193.8)
Chaharmahal and Bakhtiari		44.3 (39.6–49.2)	8.9	759 (634–879)	132 (111–153)	171 (143–198)	165.0 (137.7–191.0)
Qazvin		31.6 (30.6–32.3)	6.3	757 (629–881)	92 (76–107)	124 (103–144)	164.6 (136.6–191.5)
South Khorasan		51.0 (43.9–59.1)	10.2	747 (625–863)	171 (143–197)	186 (155–214)	162.3 (135.7–187.5)
Zanjan		30.1 (29.5–30.9)	6.0	712 (590–828)	106 (88–124)	128 (106–149)	154.6 (128.3–180.0)
Kohgiluyeh and Boyer-Ahmad		54.4 (48.4–61.9)	10.9	555 (464–640)	132 (111–153)	193 (161–222)	120.5 (100.9–139.0)
Semnan		35.1 (30.4–37.4)	7.0	537 (447–624)	116 (97–135)	143 (119–167)	116.7 (97.0–135.6)
North Khorasan		33.8 (33.4–34.6)	6.8	528 (439–614)	106 (88–123)	139 (116–162)	114.8 (95.4–133.4)
Ilam		44.6 (42.2–49.3)	8.9	436 (364–505)	118 (98–136)	170 (142–197)	94.8 (79.1–109.6)
All provinces		39.2 (26.2–87.6)	7.8	58,873 (49,024–68,287)	116 (97–135)	149 (124–173)	12,792.1 (10,652.0–14,837.6)

### Ambient PM_2.5_-related health and economic impacts at provincial- and county-level in Iran

**Figure 3:**
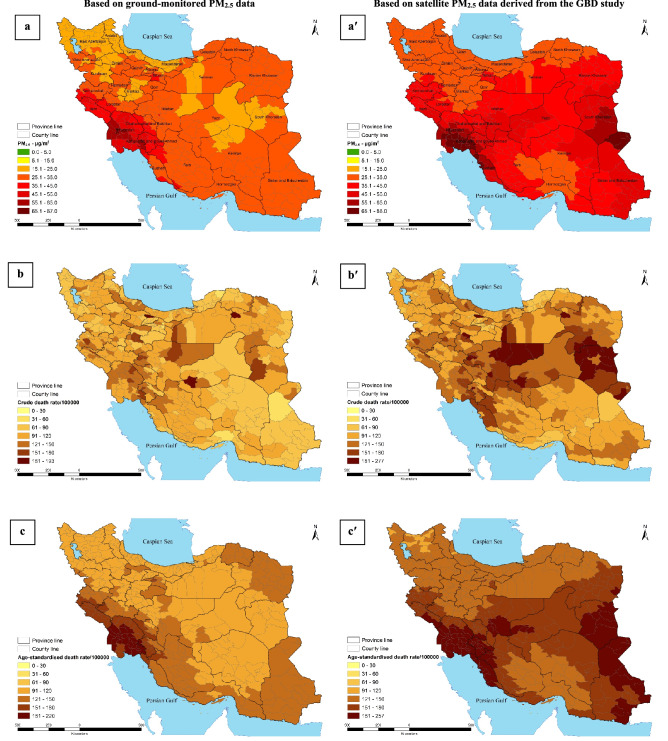
Annual PM_2.5_ mean concentrations (**a**,**a′**), crude death rate (**b**,**b′**) and age-standardized death rate (**c**,**c′**) per 10^5^ population aged ≥ 25 years old attributable to PM_**2.5**_ in all Iranian counties, 2018 (these figures were generated by ArcGIS v10.7.0.10450).

The number of provincial all cause attributable deaths due to long-term exposure to ambient PM_2.5_ based on ground and satellite-based data in 2018 is provided in Table 2. Also, the number and proportion of population aged ≥ 25 years, annual PM_2.5_ mean concentrations and their ratio to WHO AQG as well as the economic loss due to ambient PM_2.5_-related health impacts at province level are shown in Table 2. Figure 3 indicates data on the annual PM_2.5_ mean concentrations, crude death rate and age-standardized death rate per 100 000 population attributable to PM_**2**.5_ based on ground and satellite approaches in all 429 Iranian counties, 2018. As shown in Table 2 and Fig. 3a,a′, considering both measurement approaches, the population of all 429 counties experienced annual PM_2.5_ mean concentrations of higher than not only the recommendation value (5 μg m^−3^) of the new WHO's air quality guideline but also its Interim Targets 4 (10 μg m^−3^) and 3 (15 μg m^−3^). As it is evidenced in Fig. 3, both ground and satellite-based data emphasized that the population lived in western provinces (Khuzestan and Ilam) of Iran exposed to annual mean concentration of ambient PM_2.5_ exceeded even the WHO's Interim Target 1 (35 μg m^−3^). As such, the top four polluted provinces of Iran in terms of ambient PM_2.5_ mean concentrations based on ground-based data were Khuzestan (52.6 μg m^−3^), Ilam (42.7 μg m^−3^), Kohgiluyeh and Boyer-Ahmad (42.3 μg m^−3^) and Bushehr (36.3 μg m^−3^). These figures for satellite-based data changed to Khuzestan (58.4 μg m^−3^), Ilam (44.6 μg m^−3^), Kohgiluyeh and Boyer-Ahmad (54.4 μg m^−3^) and Bushehr (56.6 μg m^−3^), emphasizing no significant difference between applied approaches except for Bushehr. Based on ground and satellite-based datasets, the lowest concentration of ambient PM_2.5_ in 2018 was found in the provinces of Ardabil (22.7 versus 28.9 μg m^−3^), Gilan (23.4 and 28.7 μg m^−3^) and East Azerbaijan (23.7 and 28. 3 μg m^−3^), emphasizing a mere 5–6 μg m^−3^ difference between the applied approaches. Data provided in Table 2 and Fig. 3 reveals that the greatest differences between ground-monitored PM_2.5_ data and satellite-based ones was found for the province of Sistan and Baluchestan with 27.8 μg m^−3^, followed by South Khorasan (25.6 μg m^−3^), Bushehr (20.3 μg m^−3^), Yazd (19.3 μg m^−3^) and Isfahan (13.9 μg m^−3^). In contrast, the smallest differences between both ground-monitored PM_2.5_ data and satellite-based ones were observed in the provinces of Ilam (1.9 μg m^−3^), Qazvin (3.5 μg m^−3^), North Khorasan (3.8 μg m^−3^) and Zanjan (4.4 μg m^−3^). As shown in Table 2, considering both ground and satellite-based data, the five provinces with the highest number of all-cause mortality attributable to ambient PM_2.5_ exposures were Tehran [9899 (95% CI 8224–11,508) versus 10,515 (95% CI 8746–12,210)], Razavi Khorasan [3795 (95% CI 3149–4416) versus 4567 (95% CI 3803–5296)], Isfahan [3509 (95% CI 2909–4088) versus 5015 (95% CI 4189–5800)], Khuzestan [3375 (95% CI 2826–3893) versus 3632 (95% CI 3048–4180)] and Fars [3294 (95% CI 2736–3830) versus 3810 (95% CI 3174–4417)] and they contributed to approximately 48.4% and 46.8% of total mortality due to ambient PM_2.5_ in 2018, respectively. Applying both ground and satellite-based PM_2.5_ data, the five lowest number of attributable deaths was found in the provinces of Semnan [419 (95% CI 347–489) versus 537 (95% CI 447–624)], Ilam [430 (95% CI 359–498) versus 436 (95% CI 364–505)], South Khorasan [447 (95% CI 370–522) versus 747 (95% CI 625–863)], Kohgiluyeh and Boyer-Ahmad [459 (95% CI 383–532) versus 555 (95% CI 464–640)] and North Khorasan [489 (95% CI 406–469) versus 528 (95% CI 439–614)] and they contributed less than 3% and 5% of ambient PM_2.5_-related total mortality in Iran, respectively.

The crude death rate per 100,000 population for Iran is [97 (95% CI 81–113) based on ground-monitored PM_2.5_ data compared to 116 (95% CI 97–135) based on satellite PM_2.5_ data], nearly 50% of provinces of Iran had a higher crude death rate (Table 2; Fig. 3b,b′). The five provinces with the highest number of crude death rate per 100,000 population in 2018 in Iran due to PM_2.5_ were Khuzestan [122 (95% CI 102–140)], Ilam [116 (95% CI 97–135)], Kohgiluyeh and Boyer-Ahmad [110 (95% CI 91–127)], Kermanshah [109 (95% CI 91–127)] and Tehran [108 (95% CI 90–126)] when applying ground-based data, whereas the figures for satellite-based data changed to South Khorasan [171 (95% CI 143–197)], Isfahan [146 (95% CI 122–169)], Yazd [139 (95% CI 116–161)], Kohgiluyeh and Boyer-Ahmad [132 (95% CI 111–153)], and Chaharmahal and Bakhtiari [132 (95% CI 111–153)]. Based on the ground-based PM_2.5_ data, the lowest figures for the crude death rate per 100,000 population were estimated for the provinces of Sistan and Baluchestan [71 (95% CI 59–83)], Hormozgan [73 (95% CI 61–85)], Alborz [78 (95% CI 64–91)] and West Azerbaijan [82 (95% CI 68–95)], whereas the lowest figures for this rate when applying satellite-based PM_2.5_ data were observed in the provinces of Hormozgan [85 (95% CI 71–99)], Qazvin [92 (95% CI 76–107)], Golestan [94 (95% CI 78–110)] and West Azerbaijan [96 (95% CI 79–111)]. With regard to age-standardized death rate per 100,000 population using ground-based PM_2.5_ data (Table 2 and Fig. 3c, the highest figures were estimated for the provinces of Khuzestan [189 (95% CI 158–218)], Ilam [168 (95% CI 140–194)], Kohgiluyeh and Boyer-Ahmad [159 (95% CI 133–185)], Bushehr [140 (95% CI 117–163)] and Kermanshah [137 (95% CI 114–159)], while based on the satellite PM_2.5_ data (Table 2; Fig. 3c′) the highest figures were estimated for the provinces of Bushehr [205 (95% CI 172–236)], Khuzestan [203 (95% CI 170–234)], Sistan and Baluchestan [194 (95% CI 163–224)], Kohgiluyeh and Boyer-Ahmad Bushehr [193 (95% CI 161–222)] and South Khorasan [186 (95% CI 155–214)]. Based on both approaches, among all 429 counties, Tehran (located in Tehran province) experienced the largest attributable deaths [7802 (95% CI 6840–9072)] to ambient PM_2.5_, followed by the counties of Mashhad [1870 (95% CI 1573–2203)], Isfahan [1517 (95% CI 1258–1768)], Shiraz [1297 (95% CI 1078–1508)] and Ahvaz [939 (95% CI 788–1082)] which were located in the provinces of Razavi Khorasan, Isfahan, Fars and Khuzestan, respectively. As shown in Table 2, by far the greatest economic loss (based on the VSL) was estimated for Tehran province with 2150.9 (1787.0–2500.5) million USD, followed by the provinces of Razavi Khorasan [824.5 (95% CI 684.2–959.6)], Isfahan [762.5 (95% CI 632.1–888.2)], Khuzestan [733.3 (95% CI 614.0–846.0)] and Fars [715.7 (95% CI 594.5–832.3)].

Figure S4 shows the differences of ambient PM_**2.**5_ and the number of all cause deaths due to ambient PM_2.5_ between both approaches (ground-based data minus satellite ones) in all 429 Iranian counties, 2018. We observe some differences in the ambient PM_2.5_ and attributable deaths throughout Iran when applying both datasets. In some regions the concentrations of PM_2.5_ were lower than those reported by satellite data resulting in negative difference values. The lowest differences were observed in the western and north-west provinces and counties of Iran, whereas the greatest differences for both datasets were found in the central and eastern regions of Iran.

### Age-specific YLL and VOLY attributable to PM_2.5_ in Iran and its provinces

Table S1 gives the calculated age-specific YLL and VOLY (× 10^6^ USD) attributable to ambient PM_2.5_ exposures in Iran (2018), based on the estimated total mortality from GEMM function and IHME life expectancy table. The YLL attributable to both ground-monitored PM_2.5_ data minus satellite-based ones in Iran was 833,692 (95% CI 693,020–968,773) and 994,063 (95% CI 829,366–1,150,989) in 2018. Among the age groups, as might be expected, the highest and lowest YLL was estimated for the age group 25–29 and 80–99-year-olds, respectively. The sum of VOLY in Iran in 2018 was 3000 (95% CI 2493–3486) and 3577 (95% CI 2984–4141) million USD. On the provincial level, Tehran as the most populous province of Iran had by far the greatest YLL at all age groups [167948 (95% CI 139,763–194,946) and 178,439 (95% CI 148,677–206,873) when using ground and satellite-based PM_2.5_ data, respectively] and VOLY [604 (95% CI 503–701) and 642 (95% CI 535–744) million USD].

## Discussion

This is study is the first to estimate the health burden and economic loss due to exposure to ambient PM_2.5_ in Iran using both ground-monitored and satellite-based data. While there were some differences between the concentrations of PM_2.5_ and their related health impacts and economic loss, especially in those areas with lower number of AQMSs, the outcomes of both approaches have shown significant consistency. This should be reassuring not only to Iranian scientists and policy makers but also to those in other LMICs which lack comprehensive monitoring data and may wish to use satellite-based estimates in science and policy. Based on both PM_2.5_ datasets, the annual PM_2.5_ concentration and also its PWM were approximately 6–8 times higher than the recommendation value of the updated WHO AQGs (5 μg m^−3^)^29–31^. Our results showed that the ambient PM_2.5_ exposure concentrations had large geographical variations across Iran, suggesting a substantial spatial heterogeneity of air pollution sources in Iran, which was in agreement with previously conducted studies^16,32^. In other words, exposure to ambient PM_2.5_ concentrations varied markedly across Iranian counties with the ratio of highest to lowest annual mean nearly 3 in 2018. Among the top 100 counties with the highest annual ambient PM_2.5_, we can mostly see the counties in the provinces of Khuzestan, Ilam, Kermanshah, Lorestan, Chaharmahal and Bakhtiari, Fars, Bushehr, Tehran, and Isfahan. The underlying reasons of higher annual ambient PM_2.5_ concentrations across Iranian counties than the annual level of WHO AQGs and national standard value may be more likely due to continuing urbanization and industrialization, increasing mobile sources and associated emissions alongside ineffective ambient air quality standards and ambient air pollution abatement policies at national and subnational levels in Iran, all of which collectively amount to unsustainable development in Iran^14,15,17,32,33^. There were higher concentrations across the western and south western provinces of Iran in comparison to other parts due to dust storm events, particularly throughout the months of spring and summer seasons^16,32,34–36^. Dust storms originating from the arid regions of Iraq and Syria countries, including Tigris and Euphrates basin affect many regions of the western (Kermanshah, Ilam, and Lorestan) and south-western provinces (Khuzestan and Bushehr) of Iran^34,37,38^. Additionally, based on Hybrid Single-Particle Lagrangian Integrated Trajectory model used by previous studies in Iran, dust storm phenomenon originating each of the aforementioned sources affect central and even other provinces of Iran including Tehran, Shiraz, Isfahan, Arak, and Tabriz^39,40^. In addition to the aforementioned reasons, the ambient air quality of Khuzestan, Bushehr and Hormozgan provinces located along the bank of Persian Gulf and known as the most important industrialized provinces of Iran are also affected by other sources such as the major gas and petrochemical industries as well as ship traffics^35,41–43^. Hamoun lake located in the north part of the Sistan Basin in the southeast part of Iran acts as local source of dust storm affecting the southeast and eastern provinces and counties of Iran ^44–46^. This is consistent with the satellite-based PM_2.5_ data.

It worth to mentioned that although in the present study we focus on mortality outcomes and the vast majority deaths attributable to ambient air pollution occur in adults ≥ 25 years^25,47,48^, air pollution exposure also affects children e.g., via effects on LRI, asthma and pre-term birth and, therefore our work should not be mis-interpreted as proving a comprehensive estimate of all adverse effects of air pollution exposure.

Based on ground-monitored and satellite-based data, total mortality attributable to ambient PM_2.5_ in Iran was approximately 49,300 and 58,870 (out of 332,000 deaths recorded by the governmental organizations in our country) in 2018, respectively. YLL related to premature deaths in Iran were equivalent to the death of approximately 11 and 13 thousand children at birth. National crude death rate and age-standardized per 100 000 population attributable to ambient PM_2.5_ air pollution was 97 (95% CI 81–113) versus 116 (95% CI 97–135) and 125 (95% CI 104–145) versus 149 (95% CI 124–173) in 2018 using ground-monitored and satellite-based PM_2.5_ data, respectively. The differences between the 2 estimates could be likely due to one major factor—changes in the PM_2.5_ concentrations between the ground-monitored and satellite-based data because other used variables for estimations were the same^49^. Similar to spatial variability of ambient PM_2.5_ air pollution, PM_2.5_-related all cause and cause specific mortalities per 100,000 population had a considerable inequity throughout Iran. The observed differences are mainly due to ambient PM_2.5_ exposure levels, the distribution of the population and the proportion of population aged > 65 years in total population as the most important factor to affect mortality rate (Figure S3). Note that the ambient PM_2.5_-related all cause and cause specific mortalities per 100,000 population in the provinces with high proportion of population aged > 65 years in total population were higher than those of other counties even with the lower ambient PM_2.5_ exposure levels (Figure S3). Based on the estimate of the GBD study for our country, ambient PM_2.5_ caused approximately 39,836 (95% CI 34,477–44,833) all cause deaths for aged ≥ 25 years in 2018 (https://vizhub.healthdata.org/gbd-compare/). The difference of all cause deaths between our approach and GBD study can be attributed to the shape of concentration–response functions for all-cause of death in the GEMM and Integrated Exposure Response functions used by GBD study. Additionally, this difference is mainly due the fact that the all-cause mortality estimates in GEMM include all non-accidental deaths but the GBD estimates are based on a smaller group of causes (IHD, Stroke, COPD, LRI, LC, Diabetes and infant mortality)^23,24,47,50^. Regarding the GBD study, national crude death rate and age-standardized per 100,000 population aged ≥ 25 years attributable to ambient PM_2.5_ air pollution for Iran was 77 (95% CI 68–85) and 97 (95% CI 86–107) in 2018, respectively (http://ghdx.healthdata.org/gbd-results-tool).

In our study, the highest number of deaths was found for IHD, followed by Stroke, LRI, COPD and LC. In other words, cardiovascular and cerebrovascular disease (IHD and stroke) accounted for the majority of the deaths (nearly 61%) attributable to ambient PM_2.5_ air pollution. This is similar to the proportion of PM_2.5_-attributable mortality of IHD and stroke reported by the previous studies for China and the GBD study for the entire world^51^. The comparison among age groups and different causes of death indicates that the elderly age groups and those with cardiovascular and cerebrovascular disease are much more vulnerable and sensitive to ambient PM_2.5_ air pollution. This highlights to allocate more attention for protecting them from long- and even short-term exposure to ambient PM_2.5_. Based on our findings, the national and sub-national environmental and health authorities should much more focus on the provinces and counties with high proportion of population aged > 65 years (Figure S3) to enhance the efforts on risk management, resource allocation and air quality status.

Based on our findings and the previously conducted studies^16–18^, the residents of all provinces and counties of Iran have been, and continue to be, exposed to health-damaging levels of ambient PM_2.5_ over the three past decades. Although ambient air pollution in Iran is recognized to be a serious issue by civil society, researchers and policy-makers, there has been little progress in reducing ambient exposure, which levels remain largely unchanged (https://www.stateofglobalair.org/data/#/air/plot)^15–17^. Despite this the Iranian Department of Environment (DoE) released a revised national ambient air quality standard (NAAQS) in August 2017, in which the annual and daily PM_2.5_ standard increased (12 and 35 μg m^−3^, respectively) relative to the previous NAAQS (10 and 25 μg m^−3^), which was based on the WHO AQG 2005^18,52^. Since then, the WHO has further lowered its PM_2.5_ AQG based on the latest scientific evidence and offered guidance to governments seeking to reduce air pollution exposure and attributable mortality, including short- and long-term measures to reduce ambient air pollution and its health burden that have been successfully applied in other countries to reduce ambient air pollution-related health impacts and corresponding economic loss (https://www.who.int/news/item/22-09-2021-new-who-global-air-quality-guidelines-aim-to-save-millions-of-lives-from-air-pollution). Such approaches include shifting to clean fuels, transportation reform, reducing traffic emission(s) from vehicles and other major sources, urban landscape reform, emission trading programs, redirection of science and funding, empowering civil society, governmental and NGO-led publicity^1,13,53–57^. When part of comprehensive air quality management programs such approaches have proven effective in decreasing ambient PM_2.5_ concentrations and improving public health, but their implementation poses great challenges in many LMICs where economic development and poverty reduction have often relied on heavily-polluting economic activities^57–60^.

Our study has some limitations. Firstly, AQMSs were not widely available in all 429 counties of Iran and we inevitably used the estimated concentrations of ambient air pollutant based on neighbor stations as the proxy for population exposures, maybe leading to exposure estimation errors. Secondly, the national-level baseline mortality data was used and more elaborate data has not yet been available in Iran. Thirdly, some of the data required for health and economic burdens were borrowed/estimated from the international studies, which were mostly carried out in other countries. Another reason to do the sensitivity analysis using the GBD 2017 and 2019 exposure estimates for 2018. In terms of the relative risks borrowed to estimate health burden, the origin of ambient PM_2.5_ and its chemical and toxicological effects and even the susceptibility of population in Iran might be different from those in other countries that cohort studies have been conducted. However, to date, scientific evidence has been unable to accurately estimate the relative risk of various chemical forms or sources of ambient PM_2.5_ around all the world.

## Supplementary Information

Supplementary Information.
