# Supplementary material for: Health burden and economic loss attributable to ambient PM2.5 in Iran based on the ground and satellite data
Source: Sci Rep. 2022 Aug 23;12:14386. doi: 10.1038/s41598-022-18613-x (PMC9399101; doi:10.1038/s41598-022-18613-x)
Supplement: Supplementary file 1 — Supplementary Information. [file 41598_2022_18613_MOESM1_ESM.docx]

**Health burden and economic loss attributable to ambient PM_2.5_ in Iran based on the ground and satellit data**

Sasan Faridi ^1, 2†^, Reza Bayat ^3†^, Aaron J. Cohen ^4, 5, 6^, Ensieh Sharafkhani ^7^, Jeffrey R. Brook ^8^, Sadegh Niazi ^9^, Mansour Shamsipour ^10^, Heresh Amini ^11, 12^, Kazem Naddafi ^1, 2^, Mohammad Sadegh Hassanvand ^1, 2⁎^

^1^ Center for Air Pollution Research (CAPR), Institute for Environmental Research (IER), Tehran University of Medical Sciences, Tehran, Iran

^2^ Department of Environmental Health Engineering, School of Public Health, Tehran University of Medical Sciences, Tehran, Iran

^3^ Tehran Urban Planning & Research Center, Tehran Municipality, Tehran, Iran

^4^ Institute for Health Metrics and Evaluation, University of Washington, Seattle, Washington United States of America

^5^ Boston University School of Public Health, Boston, Massachusetts, United States of America

^6^ Health Effects Institute, Boston, Massachusetts, United States of America

^7^ School of Environment, College of Engineering, University of Tehran, Tehran, Iran

^8^ Dalla Lana School of Public Health, University of Toronto, Toronto, Ontario M5T 1P8, Canada

^9^ Queensland University of Technology (QUT), Faculty of Science, School of Earth and Atmospheric Sciences, International Laboratory for Air Quality and Health (ILAQH), Brisbane 4001, Australia

^10^ Department of Research Methodology and Data Analysis, Institute for Environmental Research (IER), Tehran University of Medical Sciences, Tehran, Iran

^11^ Department of Public Health, University of Copenhagen, Copenhagen, Denmark

^12^ Department of Environmental Health, Harvard T.H. Chan School of Public Health, Boston, MA, United States

**^† Contributed equally.^**

**^⁎^** **Corresponding Author:** MS. Hassanvand, PhD, Center for Air Pollution Research (CAPR), Institute for Environmental Research (IER) and Department of Environmental Health Engineering, School of Public Health, Tehran University of Medical Sciences, Phone: +98 88978395, Fax: +98 88978397, 8^th^ Floor, No. 1547, North Kargar Avenue, Tehran, Iran ([hassanvand@tums.ac.ir](mailto:Hassanvand@tums.ac.ir)).

**Methods**

**Study domain**

**Supplementary Text 1:**

The country; Iran; has experienced widespread outdoor PM_2.5_ air pollution challenges over the last few decades. This has been attributed to the continuing urbanization and industrialization, increasing mobile sources and associated emissions alongside ineffective ambient air quality standards and ambient air pollution abatement policies at national and sub-national levels ^1-7^. Additionally, ambient air of Iran is mostly influenced by the Middle East dust storm events and the local ones during the various seasons, particularly over the spring and summer months ^8,9^.

**Estimation of human exposure based on the ground-based and satellite PM_2.5_ data**

**Supplementary Text 2:**

It should be highlighted that the majorities of AQMSs were located in the Western, Northwestern and central provinces of Iran with largest population density. Across all AQMSs, the TEOM Filter Dynamic Measurement System method was used to measure the mass concentration of ambient PM_2.5_.

**Supplementary Text 3:**

As mentioned in the main text, we used Z scores approach to remove any inconsistency in hourly data obtained from the AQMSs. Only AQMSs with ≥50% completeness of the total hours during each year were pre-processed to reject the temporal outliers by using Z scores approach ^10,11^. Specifically, the hourly PM_2.5_ data were transformed into standard Z-scores and removed if the following three conditions were met simultaneously: **(1)** having an absolute Z score larger than 4 (|Z_t_| > 4), **(2)** the increment from the previous value being larger than 6 (Z_t_ - Z_t-1_ > 6), **(3)** the ratio of the value to its centered rolling mean of order 3 (RM3) being larger than 2 (Z_t_ / RM3 (Z_t_) > 2). By using this approach, 36 AQMSs were removed prior to next calculations. On the other hand, available AQMSs with ≥50% completeness of the total hours during each year were 111 for the year 2018. Hourly data coverage for all included AQMSs ranged from 50.2 to 97.6 percent in 2018. Note that the hourly data coverage for 36 AQMSs (out of 111 ones) was in the range of 50-70%, while the figure for the remainder was more than 70%.

**Supplementary Text 4:**

The best estimation of the PM_2.5_/PM_10_ ratio would come from a local study like the present one, as it would capture all the local meteorological and climatological conditions and sources of ambient air pollution that affect the ratio ^12^. Even in the absence of a local measurement of the ratio, a value of 0.5 could be assumed for developing countries such as Iran, and 0.65 for developed countries ^12^. In other words, the ratio of PM_2.5_/PM_10_ was calculated for each station according to the available data in the year 2018. Then the ambient PM_2.5_ concentrations were estimated based on the calculated mean of hourly PM_2.5_/PM_10_ ratios for each station. The overall mean for the PM_2.5_/PM_10_ ratios over all included stations was 0.414 (minimum, maximum and median equal to 0.342, 0.553 and 0.431) as compared to 0.33–0.44 for Delhi, 0.73 for Europe, 0.65 for America, and 0.63 for Beijing ^1,10,12,13^. The results of our ratio calculation are lower than the value of 0.50 recommended by ^12^ for developing countries likely reflecting a relatively high contribution of dust to coarse particle mass. For the cities with more than one station, the annual city-level was calculated by the hourly average air pollutant concentrations of all the validated AQMSs within each city. To evaluate the reliability of our approach to imputing the missing hourly PM_2.5_ data, we calculated the root mean square error (RMSE) for the stations with imputed data (imputed + observed) and without imputed data (just observed data) ^14,15^. In our study, the R^2^ and RMSE for measured and imputed ambient PM_2.5_ were 0.86 and 3.9 μg m^-3^ at the provincial level (**please see Figure S1 below**), demonstrating that the ambient PM_2.5_ data estimated by our method are reliable. To estimate ambient PM_2.5_ concentrations at unmonitored locations we used Voronoi Neighbor Averaging (VNA) in BenMAP-CE ^16^. VNA method initially identifies the set of AQMSs surrounding the center of population grid and then calculates PM_2.5_ concentration by taking inverse-distance weighted average (IDW) of identified monitoring stations ^14,16,17^. Hourly ambient PM_2.5_ data were used for estimation of ambient PM_2.5_ data in unmonitored counties.

|  |
| --- |

**Figure S1.** The predicted and observed values of annual mean concentration of ambient PM_2.5_ in 2018 across provinces of Iran.

**Figure S2.** Number of cause specific mortalities at various age groups due to ambient PM_2.5_ exposure in Iran (2018).

| **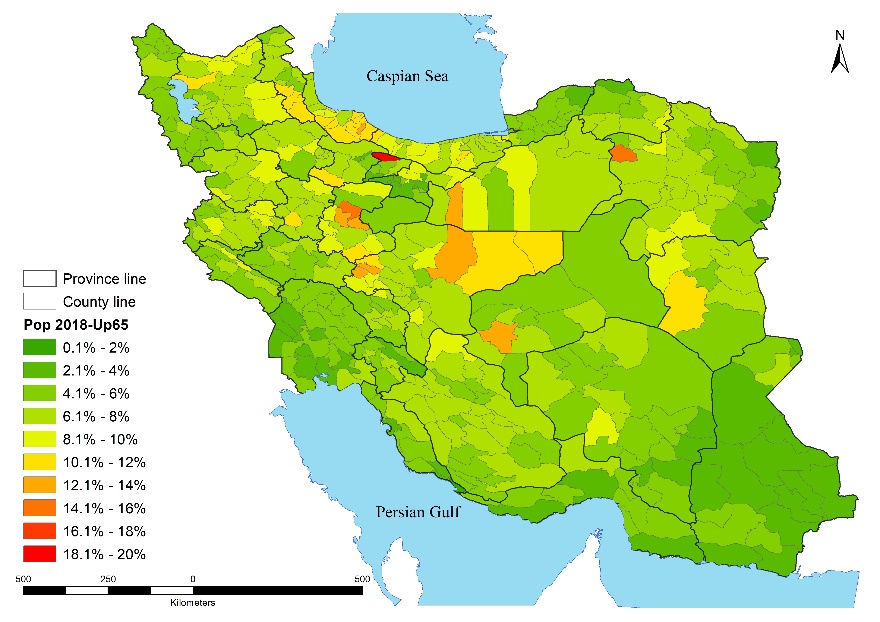** |
| --- |

**Figure S3.** The proportion of population aged > 65 years in all Iranian counties, 2018 (This figure was generated by ArcGIS v10.7.0.10450).

| 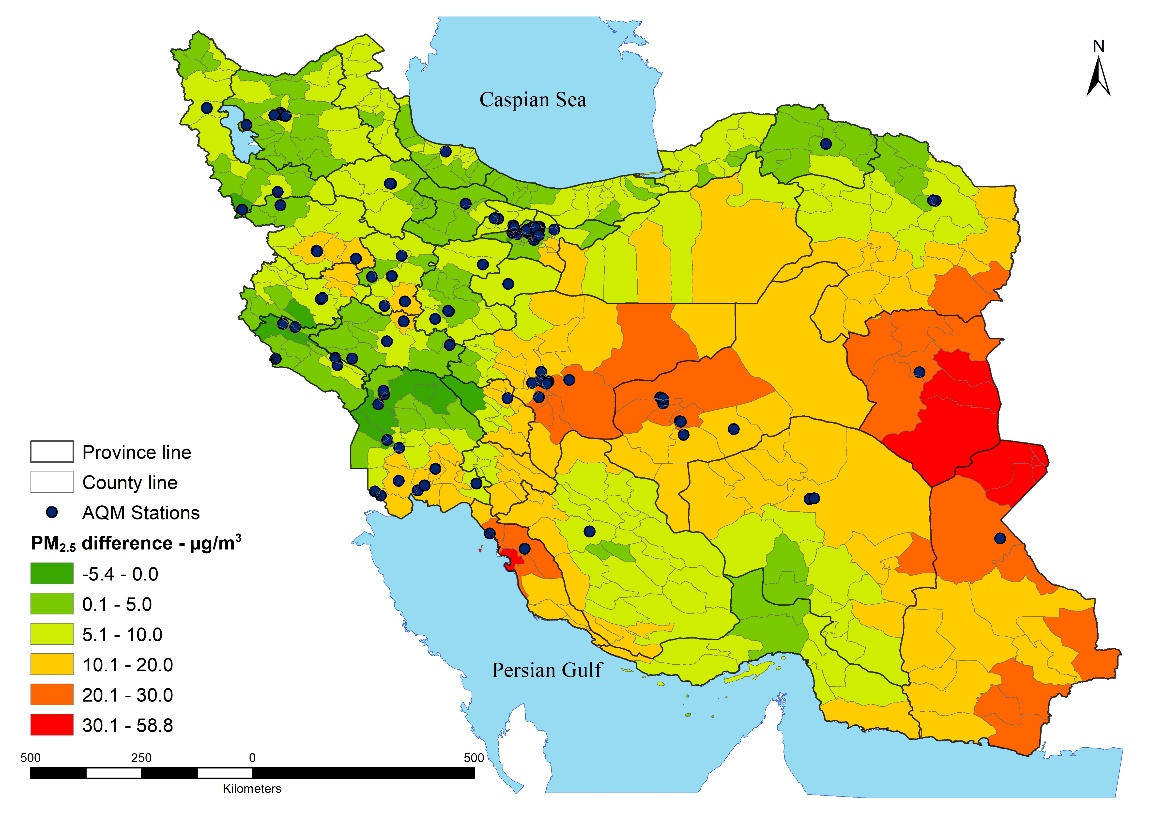 |
| --- |
| 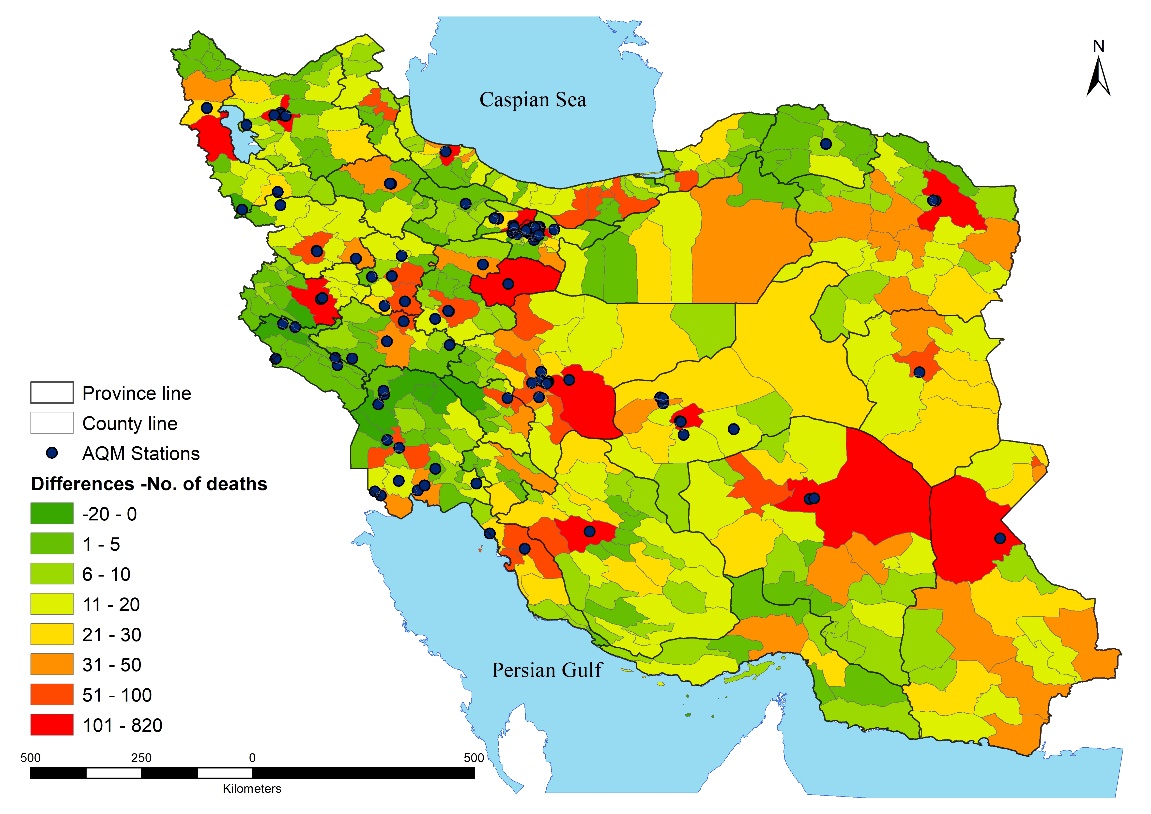 |

**Figure S4.** The differences of ambient PM_2.5_ and number of deaths between both approaches (ground-based data minus satellite ones) in all Iranian counties, 2018 (These figures were generated by ArcGIS v10.7.0.10450).

**Table S1.** Estimated age-specific YLL attributable to PM_2.5_ and its VOLY (×10^6^ US$) in Iran and its provinces in 2018 based on the ground-monitored and satellite-based PM_2.5_ data.

| Iran and its provinces | Age groups | Based on the ground-monitored data | | Based on the satellite-based data | |
| --- | --- | --- | --- | --- | --- |
|  |  | **YLL number (95% CI)** | **VOLY (95% CI)** | **YLL number (95% CI)** | **VOLY ((95% CI)** |
| Iran | **25 to 29** | 28860 (24061 – 33443) | 104 (87 – 120) | 34429 (28826 – 39731) | 124 (104 – 143) |
|  | **30 to 34** | 39448 (32882 – 45721) | 142 (118 – 165) | 47009 (39350 – 54261) | 169 (142 – 195) |
|  | **35 to 39** | 46363 (38642 – 53741) | 167 (139 – 193) | 55210 (46207 – 63736) | 199 (166 – 229) |
|  | **40 to 44** | 48688 (40582 – 56439) | 175 (146 – 203) | 57964 (48511 – 66924) | 209 (175 – 241) |
|  | **45 to 49** | 60036 (50028 – 69613) | 216 (180 – 250) | 71469 (59793 – 82547) | 257 (215 – 297) |
|  | **50 to 54** | 75032 (62497 – 87039) | 270 (225 – 313) | 89279 (74653 – 103174) | 321 (269 – 371) |
|  | **55 to 59** | 90975 (75725 – 105597) | 327 (272 – 380) | 108218 (90417 – 125149) | 389 (325 – 450) |
|  | **60 to 64** | 98452 (81879 – 114357) | 354 (295 – 411) | 117260 (97882 – 135712) | 422 (352 – 488) |
|  | **65 to 69** | 86177 (71595 – 100190) | 310 (258 – 360) | 102656 (85591 – 118929) | 369 (308 – 428) |
|  | **70 to 74** | 70752 (58719 – 82334) | 255 (211 – 296) | 84413 (70299 – 97899) | 304 (253 – 352) |
|  | **75 to 79** | 60874 (50460 – 70916) | 219 (182 – 255) | 72831 (60572 – 84568) | 262 (218 – 304) |
|  | **80 to 99** | 128033 (105950 – 149384) | 461 (381 – 537) | 153324 (127265 – 178359) | 552 (458 – 642) |
|  | **All age groups** | 833692 (693020 – 968773) | 3000 (2493 – 3486) | 994063 (829366 – 1150989) | 3577 (2984 – 4141) |
| Tehran | **All age groups** | 167948 (139763 – 194946) | 604 (503 – 701) | 178439 (148677 – 206873) | 642 (535 – 744) |
| Razavi Khorasan |  | 64361 (53496 – 74796) | 232 (192 – 269) | 77223 (64435 – 89404) | 278 (232 – 322) |
| Khuzestan |  | 60875 (51088 – 70083) | 219 (184 – 252) | 65587 (55185 – 75318) | 236 (199 – 271) |
| Isfahan |  | 57727 (47933 – 67153) | 208 (172 – 242) | 82521 (69075 – 95241) | 297 (249 – 343) |
| Fars |  | 55234 (45954 – 64128) | 199 (165 – 231) | 63705 (53169 – 73733) | 229 (191 – 265) |
| East Azerbaijan |  | 36253 (30008 – 42302) | 130 (108 – 152) | 44422 (36887 – 51672) | 160 (133 – 186) |
| Mazandaran |  | 35160 (29138 – 40979) | 127 (105 – 147) | 42109 (35006 – 48927) | 152 (126 – 176) |
| Gilan |  | 28006 (23185 – 32675) | 101 (83 – 118) | 34115 (28332 – 39678) | 123 (102 – 143) |
| West Azerbaijan |  | 27591 (22867 – 32155) | 99 (82 – 116) | 32284 (26825 – 37529) | 116 (97 – 135) |
| Kerman |  | 26123 (21674 – 30412) | 94 (78 – 109) | 34497 (28787 – 39936) | 124 (104 – 144) |
| Alborz |  | 25905 (21486 – 30169) | 93 (77 – 109) | 31943 (26596 – 37059) | 115 (96 – 133) |
| Kermanshah |  | 23232 (19335 – 26964) | 84 (70 – 97) | 26961 (22511 – 31194) | 97 (81 – 112) |
| Hamadan |  | 18109 (15020 – 21088) | 65 (54 – 76) | 21827 (18162 – 25337) | 79 (65 – 91) |
| Lorestan |  | 17399 (14458 – 20225) | 63 (52 – 73) | 20282 (16897 – 23516) | 73 (61 – 85) |
| Sistan and Baluchestan |  | 16576 (13772 – 19271) | 60 (50 – 69) | 25853 (21728 – 29722) | 93 (78 – 107) |
| Golestan |  | 16328 (13546 – 19011) | 59 (49 – 68) | 19233 (16004 – 22327) | 69 (58 – 80) |
| Kurdistan |  | 14768 (12249 – 17199) | 53 (44 – 62) | 18247 (15193 – 21170) | 66 (55 – 76) |
| Markazi |  | 14413 (11940 – 16805) | 52 (43 – 60) | 16862 (14006 – 19609) | 61 (50 – 71) |
| Hormozgan |  | 13288 (11037 – 15454) | 48 (40 – 56) | 15390 (12818 – 17846) | 55 (46 – 64) |
| Qazvin |  | 12468 (10358 – 14496) | 45 (37 – 52) | 13099 (10893 – 15217) | 47 (39 – 55) |
| Qom |  | 11606 (9638 – 13499) | 42 (35 – 49) | 13559 (11295 – 15724) | 49 (41 – 57) |
| Bushehr |  | 11169 (9304 – 12951) | 40 (33 – 47) | 16193 (13624 – 18596) | 58 (49 – 67) |
| Ardabil |  | 10922 (9044 – 12739) | 39 (33 – 46) | 13151 (10924 – 15293) | 47 (39 – 55) |
| Yazd |  | 10776 (8945 – 12539) | 39 (32 – 45) | 16041 (13433 – 18505) | 58 (48 – 67) |
| Zanjan |  | 10108 (8381 – 11775) | 36 (30 – 42) | 11489 (9547 – 13355) | 41 (34 – 48) |
| Chaharmahal and Bakhtiari |  | 9829 (8178 – 11413) | 35 (29 – 41) | 12587 (10529 – 14537) | 45 (38 – 52) |
| North Khorasan |  | 8412 (6992 – 9775) | 30 (25 – 35) | 9074 (7554 – 10530) | 33 (27 – 38) |
| Kohgiluyeh and Boyer-Ahmad |  | 7806 (6519 – 9030) | 28 (23 – 32) | 9432 (7917 – 10855) | 34 (28 – 39) |
| Ilam |  | 7484 (6259 – 8646) | 27 (23 – 31) | 7580 (6341 – 8754) | 27 (23 – 31) |
| Semnan |  | 6925 (5742 – 8068) | 25 (21 – 29) | 8872 (7390 – 10288) | 32 (27 – 37) |
| South Khorasan |  | 6888 (5709 – 8027) | 25 (21 – 29) | 11485 (9625 – 13240) | 41 (35 – 48) |

**References**

1. Shamsipour M, Hassanvand MS, Gohari K, et al. National and sub-national exposure to ambient fine particulate matter (PM2. 5) and its attributable burden of disease in Iran from 1990 to 2016. *Environmental Pollution* 2019; **255**: 113173.

2. Hadei M, Shahsavani A, Krzyzanowski M, et al. Burden of mortality attributed to PM2. 5 exposure in cities of Iran; contribution of short-term pollution peaks. *Atmospheric Environment* 2020: 117365.

3. Faridi S, Niazi S, Yousefian F, et al. Spatial homogeneity and heterogeneity of ambient air pollutants in Tehran. *Science of The Total Environment* 2019; **697**: 134123.

4. Amini H, Nhung NTT, Schindler C, et al. Short-term associations between daily mortality and ambient particulate matter, nitrogen dioxide, and the air quality index in a Middle Eastern megacity. *Environmental Pollution* 2019; **254**: 113121.

5. Amini H, Hosseini V, Schindler C, et al. Spatiotemporal description of BTEX volatile organic compounds in a Middle Eastern megacity: Tehran study of exposure prediction for environmental health research (Tehran SEPEHR). *Environmental pollution* 2017; **226**: 219-29.

6. Danaei G, Farzadfar F, Kelishadi R, et al. Iran in transition. *The Lancet* 2019; **393**(10184): 1984-2005.

7. Shahsavani A, Tobías A, Querol X, et al. Short-term effects of particulate matter during desert and non-desert dust days on mortality in Iran. *Environment international* 2020; **134**: 105299.

8. Mohammadpour K, Sciortino M, Saligheh M, Raziei T, Boloorani AD. Spatiotemporal regionalization of atmospheric dust based on multivariate analysis of MACC model over Iran. *Atmospheric Research* 2021; **249**: 105322.

9. Sotoudeheian S, Salim R, Arhami M. Impact of Middle Eastern dust sources on PM10 in Iran: Highlighting the impact of Tigris‐Euphrates basin sources and Lake Urmia desiccation. *Journal of Geophysical Research: Atmospheres* 2016; **121**(23): 14,018-14,34.

10. Faridi S, Shamsipour M, Krzyzanowski M, et al. Long-term trends and health impact of PM2. 5 and O3 in Tehran, Iran, 2006–2015. *Environment international* 2018; **114**: 37-49.

11. Yousefian F, Faridi S, Azimi F, et al. Temporal variations of ambient air pollutants and meteorological influences on their concentrations in Tehran during 2012–2017. *Scientific reports* 2020; **10**(1): 1-11.

12. Ostro B, Organization WH. Outdoor air pollution: assessing the environmental burden of disease at national and local levels: World Health Organization; 2004.

13. Maji S, Ahmed S, Siddiqui WA, Ghosh S. Short term effects of criteria air pollutants on daily mortality in Delhi, India. *Atmospheric Environment* 2017; **150**: 210-9.

14. Chen L, Shi M, Gao S, et al. Assessment of population exposure to PM2. 5 for mortality in China and its public health benefit based on BenMAP. *Environmental Pollution* 2017; **221**: 311-7.

15. Lu X, Lin C, Li W, et al. Analysis of the adverse health effects of PM2. 5 from 2001 to 2017 in China and the role of urbanization in aggravating the health burden. *Science of the Total Environment* 2019; **652**: 683-95.

16. Bayat R, Ashrafi K, Motlagh MS, et al. Health impact and related cost of ambient air pollution in Tehran. *Environmental research* 2019; **176**: 108547.

17. Manojkumar N, Srimuruganandam B. Health benefits of achieving fine particulate matter standards in India–A nationwide assessment. *Science of The Total Environment* 2020: 142999.
